# Characterization of NAC Gene Family in *Ammopiptanthus mongolicus* and Functional Analysis of *AmNAC24*, an Osmotic and Cold-Stress-Induced NAC Gene

**DOI:** 10.3390/biom14020182

**Published:** 2024-02-02

**Authors:** Tashi Dorjee, Yican Cui, Yuxin Zhang, Qi Liu, Xuting Li, Batu Sumbur, Hongxi Yan, Jie Bing, Yuke Geng, Yijun Zhou, Fei Gao

**Affiliations:** 1 Key Laboratory of Mass Spectrometry Imaging and Metabolomics, Minzu University of China, National Ethnic Affairs Commission, Beijing 100081, China; 20400268@muc.edu.cn (T.D.); 23302580@muc.edu.cn (Y.C.); 20011023@muc.edu.cn (Y.Z.); 20400256@muc.edu.cn (Q.L.); 21400267@muc.edu.cn (X.L.); songbuerbatu@muc.edu.cn (B.S.); 22302489@muc.edu.cn (H.Y.); gengyuke@muc.edu.cn (Y.G.); 2 Key Laboratory of Ecology and Environment in Minority Areas, Minzu University of China, National Ethnic Affairs Commission, Beijing 100081, China; 3 College of Life and Environmental Sciences, Minzu University of China, Beijing 100081, China; 4 College of Life Sciences, Beijing Normal University, Beijing 100080, China; bingjie@bnu.edu.cn

**Keywords:** *Ammopiptanthus mongolicus*, NAC transcription factor, AmNAC24, osmotic stress, cold stress, overexpression

## Abstract

The NAC family of transcription factors (TFs) is recognized as a significant group within the plant kingdom, contributing crucially to managing growth and development processes in plants, as well as to their response and adaptation to various environmental stressors. *Ammopiptanthus mongolicus*, a temperate evergreen shrub renowned for its remarkable resilience to low temperatures and drought stress, presents an ideal subject for investigating the potential involvement of NAC TFs in stress response mechanisms. Here, the structure, evolution, and expression profiles of NAC family TFs were analyzed systematically, and a cold and osmotic stress-induced member, *AmNAC24*, was selected and functionally characterized. A total of 86 NAC genes were identified in *A. mongolicus*, and these were divided into 15 groups. Up to 48 and 8 NAC genes were generated by segmental duplication and tandem duplication, respectively, indicating that segmental duplication is a predominant mechanism in the expansion of the NAC gene family in *A. mongolicus*. A considerable amount of NAC genes, including *AmNAC24*, exhibited upregulation in response to cold and osmotic stress. This observation is in line with the detection of numerous cis-acting elements linked to abiotic stress response in the promoters of *A. mongolicus* NAC genes. Subcellular localization revealed the nuclear residence of the AmNAC24 protein, coupled with demonstrable transcriptional activation activity. *AmNAC24* overexpression enhanced the tolerance of cold and osmotic stresses in *Arabidopsis thaliana*, possibly by maintaining ROS homeostasis. The present study provided essential data for understanding the biological functions of NAC TFs in plants.

## 1. Introduction

The maintenance of plant growth and productivity is profoundly influenced by environmental factors, with abiotic stresses such as drought, extreme temperatures, and high salinity posing significant threats to plant development and agricultural yields globally [1,2]. In response to these challenging environmental conditions, plants have evolved intricate physiological and molecular adaptation mechanisms. Plants first sense a stress signal and then transduce the stress signal to the cells through signal transduction, activating transcription factors (TFs) and further promoting the expression of stress response genes to alleviate the damage caused by stress and rebuild homeostasis under stress [3,4]. Throughout this intricate process, TFs regulate the expression of downstream genes by specifically binding to cis-acting elements in the promoters of downstream genes, playing central roles in the plant abiotic stress response [5,6].

Currently, numerous TF families have been identified as key players in the intricate regulation of plant stress responses, including NAC [7,8], WRKY [9], and MYB [10]. NAC proteins constitute a widespread TF class in diverse plant species and are named after three prototypical proteins—no apical meristem (NAM), ATAF1-2, and CUC2 (cup-shaped cotyledon)—that all feature a conserved DNA-binding domain [11,12]. The primary structure of NAC TF includes a highly conserved N-terminal DNA-binding domain and a C-terminal transcriptional regulatory domain. The N-terminal DNA-binding domain contains approximately 150–160 amino acid residues, forming a specific three-dimensional structure that can recognize and bind specific DNA sequences in the promoters of the target gene [13]. The C-terminus transcriptional regulatory domain has transcriptional activation or inhibition functions, with significant sequence variations in different NAC proteins, allowing NAC family members to regulate the expression of target genes through different mechanisms. The N-terminal DNA-binding domains can be further divided into five types, A to E, which play pivotal roles in nuclear localization, DNA elements, and functional dimer formation.

NAC family TFs have been found to play a wide range of regulatory roles in plant growth, development, and environmental stress response [14]. The overexpression of *VuNAC1* and *VuNAC2* has been shown to stimulate germination and enhance growth in *Vigna unguiculata* [15]. *ClNAC68* was reported to regulate seed development in watermelon by repressing *ClGH3.6*, a key component of growth factor signaling [16]. Accumulating evidence suggests that NAC TFs are involved in plant abiotic stress responses. In *Arabidopsis thaliana*, *ANAC019*, *ANAC055*, and *ANAC072* have been implicated in responding to high salinity and drought stress [17]. The overexpression of *OsNAC5*, *OsNAC6*, *OsNAC9*, and *OsNAC10* genes has been shown to significantly improve rice tolerance to drought stress and enhance low-temperature tolerance in rice seedlings [18,19,20,21]. Furthermore, the overexpression of specific NAC TFs has been observed to heighten tolerance to abiotic stress in *Nicotiana tabacum* [22], *Gossypium hirsutum* [23], and *Medicago truncatula* [24].

TF genes predominantly manifest in the form of gene families. The systematic analysis of the structure and evolution of TF gene families helps to gain a deeper understanding of their biological functions. The advent of high-throughput DNA sequencing technology has ushered in a rapid expansion in the sequencing of plant genomes, thereby facilitating genome-wide investigations into the NAC gene family across various plant species. So far, the extensive identification and analysis of the NAC gene family have been conducted in a range of model plants and crop species, including *A. thaliana* [25], *Oryza sativa* [26], *Vigna radiata* [27], *Capsicum annuum* [28], and *Zanthoxylum bungeanum* [29]. NAC TFs may play an important role in the environmental stress tolerance of stress-tolerant wild plants, but there is currently insufficient research on the *NAC* genes of stress-tolerant wild plants.

*A. mongolicus* is a wild evergreen broad-leaved plant in Central Asia. *A. mongolicus* has good drought and low-temperature resistance characteristics and is an important material for studying the stress tolerance mechanism of woody plants [30]. In recent years, transcriptomics, proteomics, and other omics methods have been applied to the study of the molecular mechanism of stress tolerance in *A. mongolicus* [31,32,33,34]. Some stress tolerance-related genes, including *AmCIP* [35] and *AmDREB3* [36], have been isolated and functionally studied. However, a systematic identification and characterization of the TF gene families associated with the abiotic stress of *A. mongolicus* are still lacking. A genome-wide analysis of the NAC gene family would help to enhance the understanding of the evolution and biological functions of NAC TFs in *A. mongolicus*. Although the *AmNAC11* [37] and *AmNTL1* [38] (a NAC gene) of *A. mongolicus* have been isolated and functionally studied, a comprehensive analysis and systematic identification of the entire *A. mongolicus* NAC (AmNAC) family has not yet been reported.

In the present study, we identified the NAC family TFs in *A. mongolicus* and analyzed their protein and gene structures, phylogenetic evolution, gene amplification, and gene expression patterns. *AmNAC24*, a cold and osmotic-stress-induced NAC gene, was selected and functionally characterized. This study provides important data for understanding the biological roles of the NAC gene family in *A. mongolicus* under environmental stress.

## 2. Materials and Methods

### 2.1. Identification and Characterization of the AmNAC TF Family

The genome data of *A. mongolicus* was obtained from our laboratory and the assembled genome has been deposited in GenBank under accession PRJNA1067861. The gene model of the NAC domain was obtained from the Pfam (http://pfam.xfam.org/, accessed on 22 March 2023) database with entry number PF02365. Based on the PF02365 model with a threshold of e-value ≤10^−5^, HMMER3.0 [39] was used to search the protein data of *A. mongolicus*. The NAC protein sequences of *A. thaliana* were obtained from PlantTFDB (http://planttfdb.gao-lab.org/, accessed on 23 March 2023) and subjected to a local BLASTp program against the *A. mongolicus* genome, using a threshold e-value of ≤10^−5^. The union of the two preliminary identification results was submitted to the Pfam, CDD (https://www.ncbi.nlm.nih.gov/, accessed on 15 April 2023), and SMART (http://smart.embl-heidelberg.de/, accessed on 15 April 2023) databases to check whether NAC candidate proteins have NAC conserved domains. The basic physicochemical properties of the AmNAC protein, including its amino acid composition, molecular weight (MW), isoelectric point (pI), and grand average of hydropathicity, were analyzed using the ProtParam (https://web.expasy.org/protparam/, accessed on 20 April 2023) tool. The subcellular localization of AmNAC proteins was predicted using the PSORT tool (https://www.genscript.com/wolfpsort.html, accessed on 22 April 2023).

### 2.2. Chromosomal Location of AmNAC Genes

All *AmNAC* genes were mapped to 9 chromosomes using TBtools v1.0 (https://github.com/CJ-Chen/TBtools, accessed on 5 May 2023) [40] based on the GFF file (genome annotation) of the *A. mongolicus* genome. All *AmNAC* genes were named according to the position of the *AmNAC* genes on the chromosomes, and the naming method involved sequentially numbering them from the top to the bottom along the chromosomes.

### 2.3. Multiple Sequence Alignment, Phylogenetic Analysis, Gene Structure, and Motif Composition

MAFFT v7 (https://mafft.cbrc.jp/alignment/software/, accessed on 20 April 2023) [41] was used to perform multiple sequence alignment. A phylogenetic tree was constructed using the neighbor-joining method with 1000 bootstrap replicates. This phylogenetic tree was also visualized using the Evolview (https://www.evolgenius.info/, accessed on 22 April 2023) program. MEME (http://MEME-suite.org/, accessed on 25 April 2023) [42] was utilized to detect the conserved motif of the AmNAC protein with the following parameters: the maximum length of the recognition motif was 10, the minimum width of the motif was 6, and the maximum width was 50. NCBI batch CD-Search (https://www.ncbi.nlm.nih.gov/cdd, accessed on 30 April 2023) was used to obtain domain information. The 1500 bp upstream sequence of each *AmNAC* gene was extracted and submitted to the PlantCARE [43] program (http://bioinformatics.psb.ugent.be/webtools/PlantCARE/html/, accessed on 3 May 2023) for cis-regulatory element prediction.

### 2.4. Gene Duplication and Synteny Analysis of AmNACs

The protein sequence of *A. mongolicus* was used for self-blast alignment with e-value ≤ 1 × 10^−5^. MCScanX v1.1.11 (https://github.com/wyp1125/MCScanX, accessed on 8 May 2023) [44] was used to obtain collinearity and tandem files, and the segmental and tandem duplication genes of *AmNACs* were screened from this result. The genome data of *Vitis vinifera*, *A. thaliana*, *M. truncatula*, and *Lupinus albus* were obtained from the Phytozome v13 (https://phytozome-next.jgi.doe.gov/, accessed on 10 May 2023) and NCBI (https://www.NCBI.nlm.nih.gov/, accessed on 10 May 2023) databases. Synteny analysis between species was performed using MCScanX v1.1.11 (https://github.com/wyp1125/MCScanX, accessed on 8 May 2023), and the synteny maps were visualized with TBtools v1.0 (https://github.com/CJ-Chen/TBtools, accessed on 11 May 2023).

### 2.5. Ka and Ks Calculation

The values for non-synonymous (Ka) and synonymous (Ks) substitutions in duplicated *AmNAC* gene pairs were determined using the KaKs calculator 2.0 (https://sourceforge.net/projects/kakscalculator2/, accessed on 20 May 2023) [45]. The selection pressure on these gene pairs was inferred by estimating the ratio of Ka to Ks. The duplication time of homologous genes within the *A. mongolicus* NAC gene family was calculated using the formula T = Ks/2λ.

### 2.6. Expression Analysis of AmNAC Genes Using Transcriptome Sequencing Data

The transcriptomic data of *A. mongolicus* from summer to winter were collected from the NCBI database (SRR16479821–SRR16479829). TopHat v2.2.1 (http://ccb.jhu.edu/software/tophat/, accessed on 12 June 2023) [46] was applied to map the reads to the genome of *A. mongolicus*. The FPKM value was utilized to estimate the gene expression levels, and the heat maps representing these expression levels were generated using TBtools v1.0 (https://github.com/CJ-Chen/TBtools, accessed on 15 June 2023). Hierarchical clustering analysis was conducted via the OmicShare platform (https://www.omicshare.com/tools/, accessed on 24 May 2023).

### 2.7. Plant Materials and RNA Extraction

The seed samples were collected from Ordos City, Inner Mongolia Autonomous Region, China. The seed germination and planting conditions were based on a previous study [47]. Three-month-old *A. mongolicus* seedlings were used for the control check (CK), osmotic stress (OT), drought stress (DT), and cold stress (CT) treatment. For the osmotic stress treatment, 60 seedlings were irrigated with 20% PEG 6000 solution for 0 h, 6 h, 24 h, 72 h, and 7 d. For the drought stress treatment, 15 seedlings plants were placed under natural drought conditions for 3 weeks. For the cold stress treatment, 60 seedlings were moved to a growth chamber and cultured at 4/15 °C for 0 h, 6 h, 24 h, 72 h, and 7 d. Leaf samples were collected from each time-point, immediately frozen with liquid nitrogen and stored at −80 °C until RNA extraction. Total RNA was isolated from the tissues of *A. mongolicus* using the Trizol reagent, adhering to the guidelines provided by the manufacturer (Invitrogen, Carlsbad, CA, USA). Subsequently, reverse transcription was performed using a FastQuant RT Kit (with gDNase) (TIANGEN, Beijing, China).

### 2.8. qRT-PCR Analysis

Sixteen *AmNAC* genes and eight genes related to reactive oxygen species (ROS) activity were selected for qRT-PCR validation. The first strand cDNA was produced by the reverse transcription of RNA from different treatment groups. Gene-specific primers and the internal control (Actin) primers were designed with primer premier 5.0 (Table S1). qRT-PCR was carried out with the protocol outlined in a former investigation [48].

### 2.9. Vector Construction, Arabidopsis Transformation, and Transgenic Plant Materials

The coding region of the *AmNAC24* gene was isolated and ligated into vector pCAMBIA1305 using the endonuclease XbaI and BamHI. The construct was transformed into the *A. tumefaciens* strain GV3101, and then transformed into *A. thaliana* through the floral-dip method. Hygromycin (25 µg/mL) was used to screen transgenic plants. After sterilization, wild-type (WT) plants and two transgenic T2 lines were seeded on Murashige and Skoog (MS) agar plates. The plants flourished in a controlled greenhouse environment, maintaining a temperature of 22 °C, a steady light intensity of 400 µmol·m^−2^·s^−1^, and following a 16/8 h (light/dark) cycle.

### 2.10. Subcellular Localization and Transactivation Assay of AmNAC24

The transcriptional activity of *AmNAC24* was detected using a yeast expression system. The coding sequence of *AmNAC24* was isolated and linked to the pGBKT7 vector. The resulting pGBKT7-*AmNAC24* vector and pGBKT7 (vector control) were transferred into AH109 yeast cells, and the positive clones were screened on the media lacking tryptophan (SD/-Trp) or tryptophan and histidine (SD/-Trp/-His). Then, the positive clones were transferred to the medium with X-gal (SD/-Trp/-His/X-gal) and cultured in darkness for 3–4 days until color development. Transcriptional activation activity was evaluated based on the growth status of different transformants.

### 2.11. Evaluation of the Tolerance of Transgenic Arabidopsis to Osmotic Stress and Cold Stress

Seeds of WT *A. thaliana* and two lines of transgenic *A. thaliana* were germinated on MS plates. Seedlings with a similar root length (2–3 mm) were moved to MS medium supplemented with 200 mM of mannitol to simulate osmotic stress, or moved to a growth chamber and cultured at 15 °C to simulate cold stress. The root lengths of the seedling in each group were measured to evaluate the tolerance of transgenic *Arabidopsis* to cold stress and osmotic stress.

### 2.12. Physiological Parameter Measurements

The levels of malondialdehyde (MDA) and proline were measured using the Solarbio MDA detection kit (Solarbio, Beijing, China) and Solarbio proline detection kit (Solarbio, Beijing, China). The activities of peroxidase (POD), ascorbate peroxidases (APX), superoxide dismutase (SOD), and catalase (CAT) were determined in accordance with the methodologies described in a previous study [49]. Diamindbenzidine (DAB) and nitroblue tetrazolium (NBT) staining was conducted according to a previously described method [50].

## 3. Results

### 3.1. Identification of AmNAC Proteins in A. mongolicus and Their Physicochemical Properties and Chromosomal Locations

**Figure 1:**
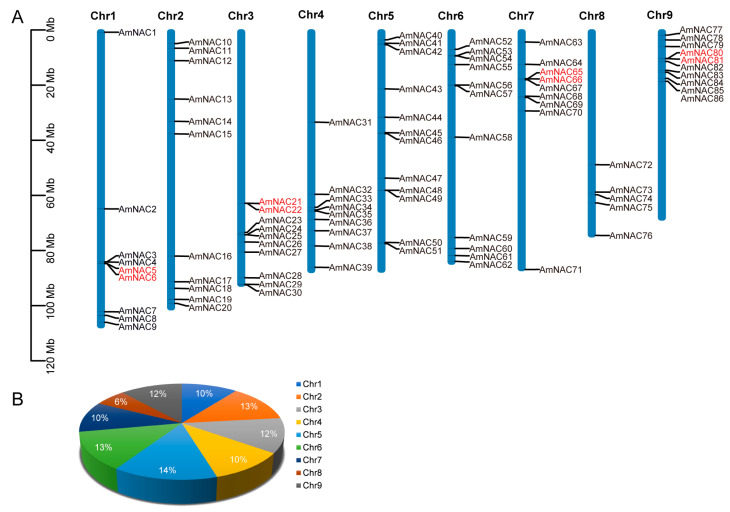
Chromosomal location of the NAC family genes in the *A. mongolicus* genome. (**A**) Chromosomal localization of *AmNAC* genes; *AmNAC* genes are shown in black, and genes in red font represent tandem duplicated genes. (**B**) Pie chart representing the number of *AmNAC* genes on each chromosome.

A total of 86 AmNAC TFs were identified and named *AmNAC1*-*AmNAC86* based on their chromosomal location (Figure 1A). The predicted MW ranged from 10.43 to 76.84 kDa (Table S2). Notably, AmNAC6 had the longest protein sequence length (671 AA) and the largest protein molecular weight (76.84 kDa), while AmNAC40 had the shortest protein sequence length (89 AA) and the smallest protein molecular weight (10.43 kDa). The pI ranged from 4.48 (AmNAC3 and AmNAC4) to 10.53 (AmNAC21), with an average of 6.65. Among them, there were 55 acidic proteins and 31 alkaline proteins. The values of the grand average of hydropathicity indicate that all AmNAC genes are hydrophobic. Subcellular localization predictions showed that 91.86% of AmNAC members were localized to the nucleus.

All nine chromosomes of *A. mongolicus* contained *NAC* genes (Figure 1A). Among them, Chr 5 had the highest number of *NAC* genes distributed, reaching 12 and accounting for 14% of the total number of *NAC* genes (Figure 1B), while Chr 8 had the lowest number of *NAC* genes, with only 5.

### 3.2. Multiple Sequence Alignment and Phylogenetic Analysis of the AmNACs

To investigate the sequence characteristics of the conserved regions of AmNAC proteins, multiple sequence alignment was performed on all the NAC proteins. Most of the N-terminals of AmNAC proteins have five conserved motifs, i.e., motif A~motif E (Figure S1). However, certain NAC members do not contain all five motifs; for example, AmNAC3 and AmNAC4 only contain motif A and B, AmNAC2 lacks motif A, and AmNAC33 lacks motif D and E. Some AmNAC proteins have an incomplete motif; for example, AmNAC2, AmNAC3, and AmNAC4 proteins have an incomplete motif B, and AmNAC46 and AmNAC73 proteins have an incomplete motif C. The variation in motif distribution reflects the functional diversity of the AmNAC family members in *A. mongolicus*.

**Figure 2:**
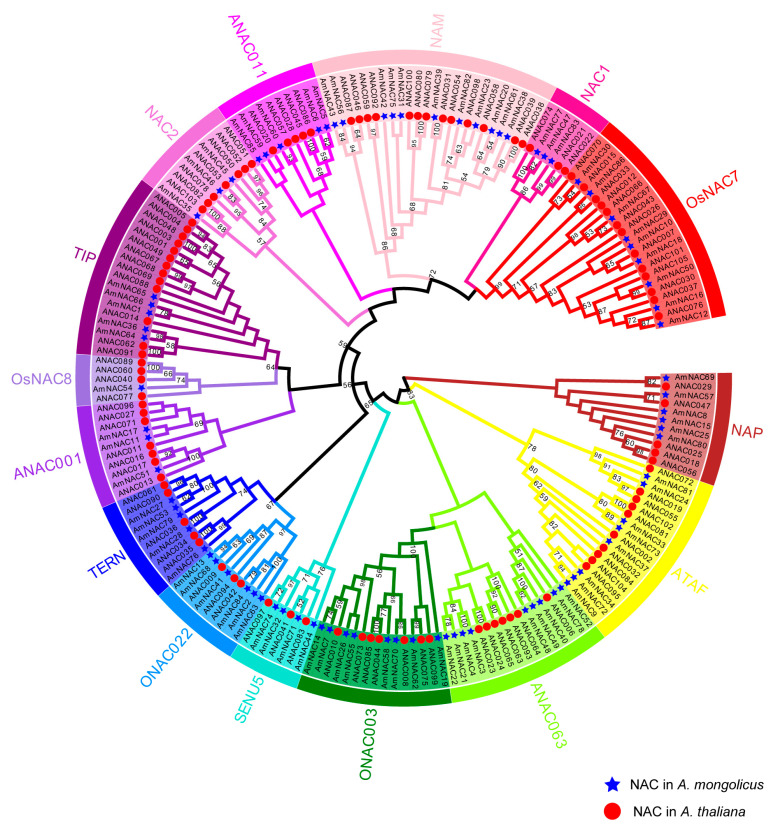
Phylogenetic tree of NAC TF families in *A. mongolicus* and *A. thaliana*. Different colors represent different subfamilies, with red circles representing the NAC members of *A. thaliana* and blue stars representing the NAC members of *A. mongolicus*.

To investigate the evolutionary relationships of AmNAC gene families, a phylogenetic tree was constructed using the sequences of 86 NACs in *A. mongolicus* and 105 NACs in *A. thaliana*. Based on the annotation of *A. thaliana* NAC proteins, all AmNAC proteins were divided into 15 groups (Figure 2). Among the 15 groups, the NAM subfamily was the largest, containing 11 members, followed by group OsNAC7 and group ONAC003, with 9 members each. The OsNAC8 group is the smallest group, containing only one AmNAC member.

### 3.3. Analysis of AmNAC Gene Structure and Motif

To investigate the gene structure diversity of AmNAC family genes, the conserved motifs and intron distributions were analyzed. In total, 10 conserved motifs (Figure S2A) were identified within the AmNAC family and most of the members contained motif 1, motif 2, motif 3, motif 4, and motif 6 at the N-terminal (Figure S2B). Specific protein motifs were exclusive to certain subfamilies. For example, motif 7 and motif 8 only exist in the AmNAC011 subfamily, and motif 9 only exists in the NAM and ATAF subfamilies (Figure S2B). The intron distribution of the sequences of the AmNAC family genes showed that 86 *AmNAC* genes contained 1 to 8 introns with varying numbers (Figure S2C). *AmNAC65*, which has the largest number of introns, has eight introns, while *AmNAC3* and *AmNAC4* have only one intron.

### 3.4. Cis-Acting Element Present in Promoters of the AmNAC Family Genes

**Figure 3:**
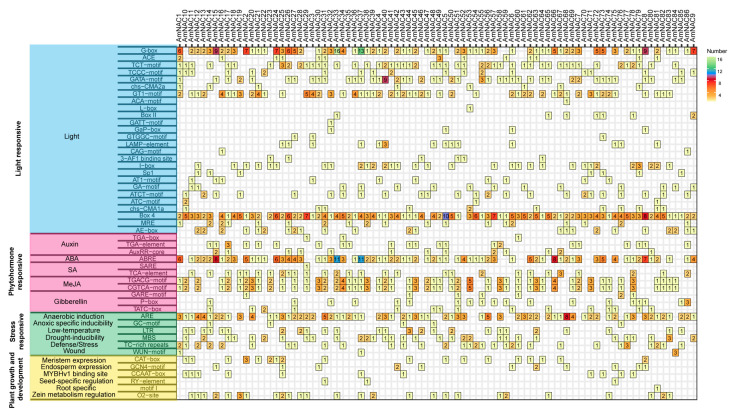
The number of detected cis-acting elements in the promoter region of *AmNAC* genes. The colors of the box ranging from yellow to green represent the number of homeopathic elements. The different color modules on the left represent different cis-acting element categories.

To investigate the potential expression patterns of *AmNAC* genes, cis-acting elements were predicted for the 1500 bp sequence upstream of the *AmNAC* genes. Forty-nine distinct cis-elements were identified, falling into four distinct categories: light responsive, phytohormone responsive, stress responsive, and plant growth and development related (Figure 3). Among the four categories, light-responsive cis-acting elements are the most abundant, with 26 cis-acting elements; this is followed by phytohormone-responsive cis-acting elements, with 11 cis-acting elements. In the category of light responsiveness, Box 4 emerged as the predominant element, with G-box being the subsequent major component. In the category of phytohormone responsiveness, the TGACG motif and CGTCA motif associated with elements responsive to methyl jasmonate (MeJA) were the most abundant, followed by the TCA motif associated with auxin-responsive elements. The cis-acting elements related to plant growth and development were mainly dominated by the O2-site and CAT-box related to zein metabolism regulation and meristem expression (Figure 3). In the category of stress responsiveness, the ARE element was the most abundant, followed by TC-rich repeats, MBS, and LTR, which are associated with defense and stress responsiveness, drought-inducibility and low-temperature responsiveness. The presence of these cis-acting elements highlighted the possible roles of *AmNAC* genes in plant growth, development and response to environmental stress.

### 3.5. Gene Family Evolution and Comparative Genomic Analysis

**Figure 4:**
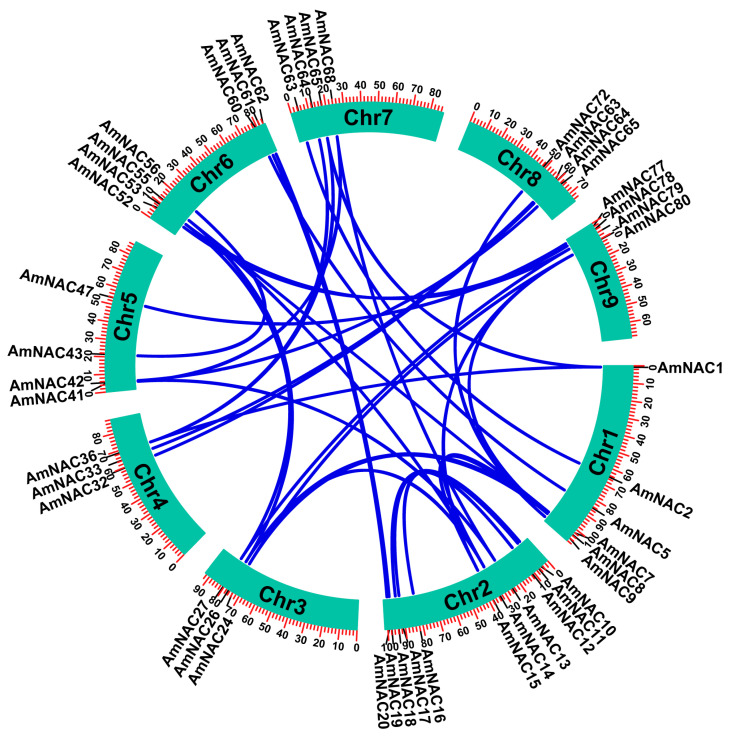
The segmentally duplicated *AmNAC* genes and their syntenic relationship. All *AmNAC* gene pairs are marked according to their chromosome distribution in the *A. mongolicus* genome, and blue lines indicate segmental duplication events associated with the *AmNAC* genes.

To investigate the mechanism of gene amplification in the NAC gene family of *A. mongolicus*, the synteny of *AmNAC* genes was analyzed. In total, 38 paralogous gene pairs were identified among 86 *AmNACs*, containing 54 *AmNAC* genes. All identified gene pairs were generated by segmental duplication or tandem duplication events occurring in the evolution of the *A. mongolicus* genome. Predominantly, the amplification events were attributed to segmental duplication (46 genes), while a smaller subset resulted from tandem duplication (8 genes) (Figure 1A). The segmentally duplicated *AmNAC* genes were distributed on every chromosome, with the highest number on chromosome 2, containing 11 *AmNAC* genes, and the lowest quantity on chromosomes 3 and 4, with only 3 genes each (Figure 4). The tandemly duplicated *NAC* genes were distributed in chr1, 3, 7, and 9 (Figure 1A).

The Ka/Ks metric serves as a tool for identifying selection pressures in gene duplication events. The analysis of all 38 duplicated gene pairs revealed Ka/Ks ratios consistently < 1 (Table S3), indicating a prevailing trend of gradual evolution among most *AmNAC* genes. This observation suggests that these genes likely underwent purifying selection throughout their evolutionary trajectory. The KaKs values for all segmental repeat pairs varied between 0.05 and 0.42, averaging at 0.20, while those for tandem repeat gene pairs fluctuated between 0.13 and 0.63, with an average of 0.41. The Ks values were further used to estimate the divergence time of the 38 duplication gene pairs, which ranged from 0.1–1.4 million years ago (Mya). The oldest gene duplication event occurred at approximately 1.41 Mya in a segmental duplication gene pair (*AmNAC1*–*AmNAC36*), and the most recent gene duplication event occurred at 0.06 Mya in a tandem duplication gene pair (*AmNAC5*–*AmNAC6*).

To identify the evolutionary relationships of *AmNAC* genes among different plant species, synteny analyses were performed between *A. mongolicus* and *V. vinifera*, *A. thaliana*, *M. truncatula*, and *L. albus*. In total, 76, 49, 71, and 73 *AmNAC* orthologs were identified in *A. mongolicus* vs. *V. vinifera*, *A. mongolicus* vs. *A. thaliana*, *A. mongolicus* vs. *M. truncatula*, and *A. mongolicus* vs. *L. albus*, respectively (Figure S3).

### 3.6. Expression Patterns of A. mongolicus AmNAC Genes from Summer to Winter

**Figure 5:**
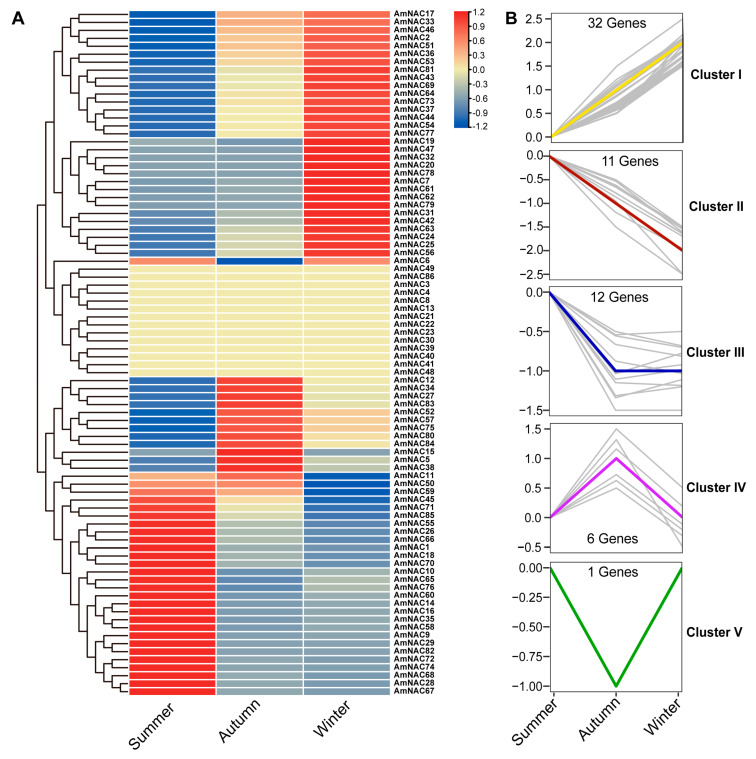
Expression patterns of *AmNAC* genes from summer to winter based on transcriptome data. (**A**) Heatmap indicating the expression levels of *AmNAC* genes from summer to winter; red to blue color represents gene expression from high to low. (**B**) Expression trend observed in *AmNAC* genes from summer to winter.

To investigate the expression patterns of *AmNAC* genes from summer to winter, the transcription levels of *AmNAC* genes in different seasons were analyzed based on transcriptome data. Compared to summer, seven *AmNAC* genes exhibited significantly increased expression in autumn, while six showed significant decreases. Compared to summer, nine *AmNAC* genes demonstrated significantly increased expression in winter, whereas five exhibited significant decreases (Figure 5A).

The expression trend of *AmNAC* genes from summer to winter were further elucidated through cluster analysis. The expression trends of the 62 *AmNAC* genes from summer to winter were classified into five clusters: I, II, III, IV, and V (Figure 5B). Cluster I, containing 32 *AmNAC* genes including *AmNAC24*, *AmNAC51*, and *AmNAC81*, demonstrated a gradual increase in expression from summer to winter. In contrast, cluster II, containing 11 *AmNAC* genes including *AmNAC1*, *AmNAC55*, and *AmNAC71*, displayed a gradual decrease in expression. The expression levels of the 12 genes in Cluster III decreased in summer and autumn, but no significant difference was detected between autumn and winter.

### 3.7. Expression Patterns of AmNAC Genes under Osmotic and Cold Stresses

**Figure 6:**
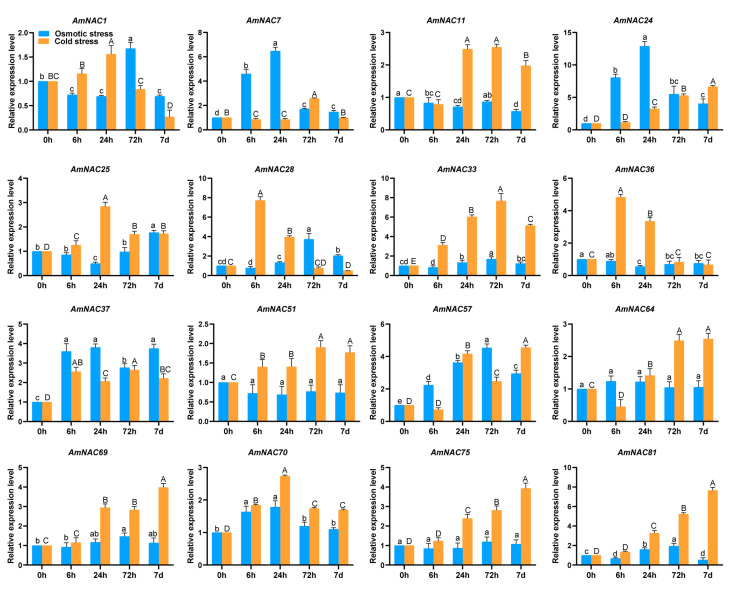
Expression patterns of 16 *AmNAC* genes under osmotic stress and cold stress based on qRT-PCR analysis. The blue bar graph represents the gene expression levels under osmotic stress, and the orange bar graph represents the gene expression levels under cold stress. The statistical significance of different treatments is distinguished by uppercase and lowercase letters, and the same letter indicates that the difference is not significant.

To determine the expression patterns of *AmNAC* genes under osmotic and cold stresses, the expression patterns of 16 randomly selected *AmNAC* genes under osmotic and cold stress were analyzed by qRT-PCR (Figure 6). Among the 16 *AmNAC* genes, 5 exhibited significant upregulation in response to osmotic stress, including *AmNAC1* and *AmNAC7*, and 2 genes displayed significant downregulation under osmotic stress, including *AmNAC33* and *AmNAC36*; 14 genes were significantly upregulated under cold stress, including *AmNAC24* and *AmNAC81*, while no *AmNAC* genes were significantly downregulated under cold stress. It is worth noting that a total of seven *AmNAC* genes were upregulated under both osmotic stress and cold stress, including *AmNAC24*, *AmNAC37*, and *AmNAC57*.

### 3.8. Subcellular Localization and Transcriptional Activation Activity of AmNAC24 Protein

**Figure 7:**
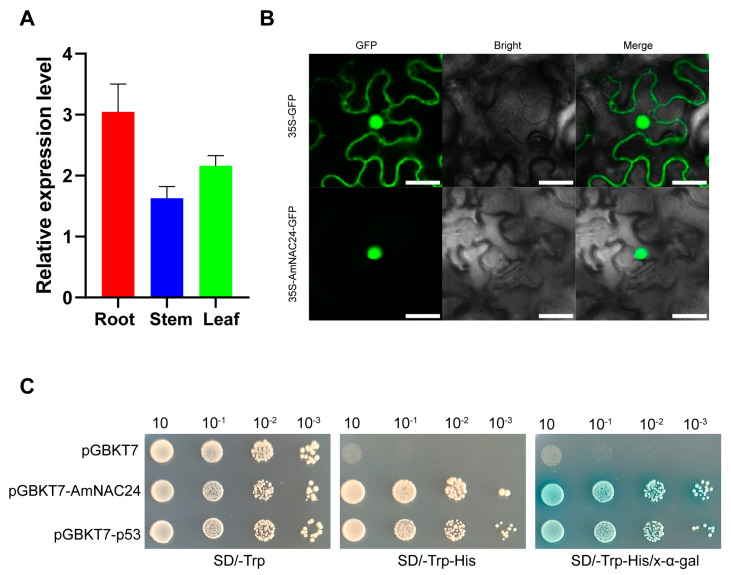
Subcellular localization and transcriptional activation activity analysis of AmNAC24 protein. (**A**) Expression of *AmNAC24* in roots, stems, and leaves of *A. mongolicus*. (**B**) Subcellular localization analysis was performed by the transient expression of the *AmNAC24* gene in tobacco, bars = 50 μm. (**C**) Determination of the transcriptional activation ability of *AmNAC24* using the yeast expression system. Yeast cells expressing pGBKT7 empty were used as negative controls, while yeast cells expressing pGBKT7-p53 were used as positive controls.

The *AmNAC24* gene is expressed in roots, stems, and leaves (Figure 7A). To determine the subcellular localization of AmNAC24, the coding sequence of the *AmNAC24* gene was fused to GFP and transiently expressed in tobacco. It was found that the GFP fluorescent signals diffused in tobacco cells, and that the green fluorescent signal of the fused *AmNAC24*-GFP was only observed in the nucleus of tobacco cells (Figure 7B), indicating that AmNAC24 is a nuclear-localized protein. To determine the transcriptional activation activity of the AmNAC24 protein, yeast cells were transformed with the fusion construct pGBKT7-AmNAC24, pGBKT7 (negative control), and pGBKT7-p53 (positive control). Yeast cells containing pGBKT7-AmNAC24 or pGBKT7-p53 grew well in SD/-Trp/-His/x-α-gal medium, while yeast cells containing the negative control pGBKT7 did not grow (Figure 7C), indicating that AmNAC24 has transcriptional activation activity in yeast.

### 3.9. Overexpression of AmNAC24 Gene Enhanced the Tolerance of A. thaliana to Osmotic Stress

**Figure 8:**
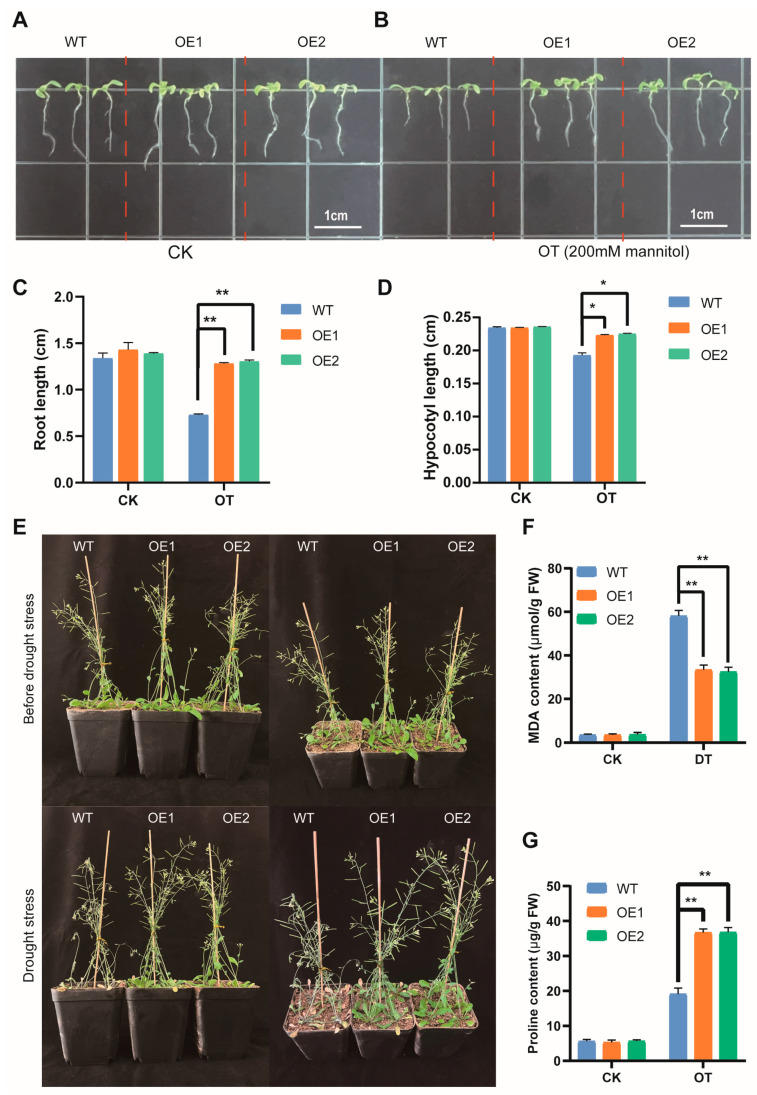
Comparison of root and hypocotyl growth of the two *AmNAC24* overexpression lines and WT plants under osmotic stress. (**A**) WT, *AmNAC24 OE1*, and *AmNAC24 OE2* plants cultured at normal conditions. (**B**) WT, *AmNAC24 OE1*, and *AmNAC24 OE2* plants under osmotic stress. (**C**) Root length of WT, *AmNAC24 OE1*, and *AmNAC24 OE2* plants in CK and OT. (**D**) Hypocotyl length of WT, *AmNAC24 OE1*, and *AmNAC24 OE2* plants in CK and OT. (**E**) Phenotypes of WT, *AmNAC24 OE1*, and *AmNAC24 OE2* plants under normal and natural drought conditions. (**F**) MDA content of WT, *AmNAC24 OE1*, and *AmNAC24 OE2* plants measured under drought conditions. (**G**) Proline content of WT, *AmNAC24 OE1*, and *AmNAC24 OE2* plants measured under drought conditions. Each experiment was performed in three independent biological replicates. Values are expressed as mean ± standard deviation, and the LSD method was used to evaluate the significant difference, * *p* < 0.05, ** *p* < 0.01.

To investigate the function of *AmNAC24* in the tolerance of plants to osmotic stress, the *AmNAC24* gene was introduced into *A. thaliana*, and the phenotypic differences between the WT plants and the two transgenic lines (*AmNAC24 OE1* and *AmNAC24 OE2*) were observed under osmotic stress conditions (20% PEG 6000/22 °C). Seedlings of the WT plants and the two transgenic lines showed a similar growth performance under normal conditions (Figure 8A). Under osmotic stress, the WT plants and transgenic lines (*OE1* and *OE2*) grew slower than the seedlings grown under normal conditions (Figure 8B). However, the measurements of the root and hypocotyl length indicated that the *A. thaliana* seedlings of two *AmNAC24*-overexpressing lines exhibited better growth than that of the WT plants (Figure 8C,D). We also conducted drought tolerance experiments using 2-month-old *Arabidopsis* plants. Transgenic lines overexpressing *AmNAC24* exhibited superior growth compared to WT plants when subjected to natural drought conditions (Figure 8E). Transgenic plants exhibited a notably reduced MDA content compared to WT plants when subjected to drought stress. Conversely, there was no statistically significant disparity between WT and transgenic plants under normal conditions (Figure 8F). Compared with the WT plants, the analysis of the leaf proline content revealed a significantly higher accumulation of proline in the OE plants under drought conditions (Figure 8G).

### 3.10. Overexpression of AmNAC24 Gene Enhanced the Tolerance of A. thaliana to Cold Stress

**Figure 9:**
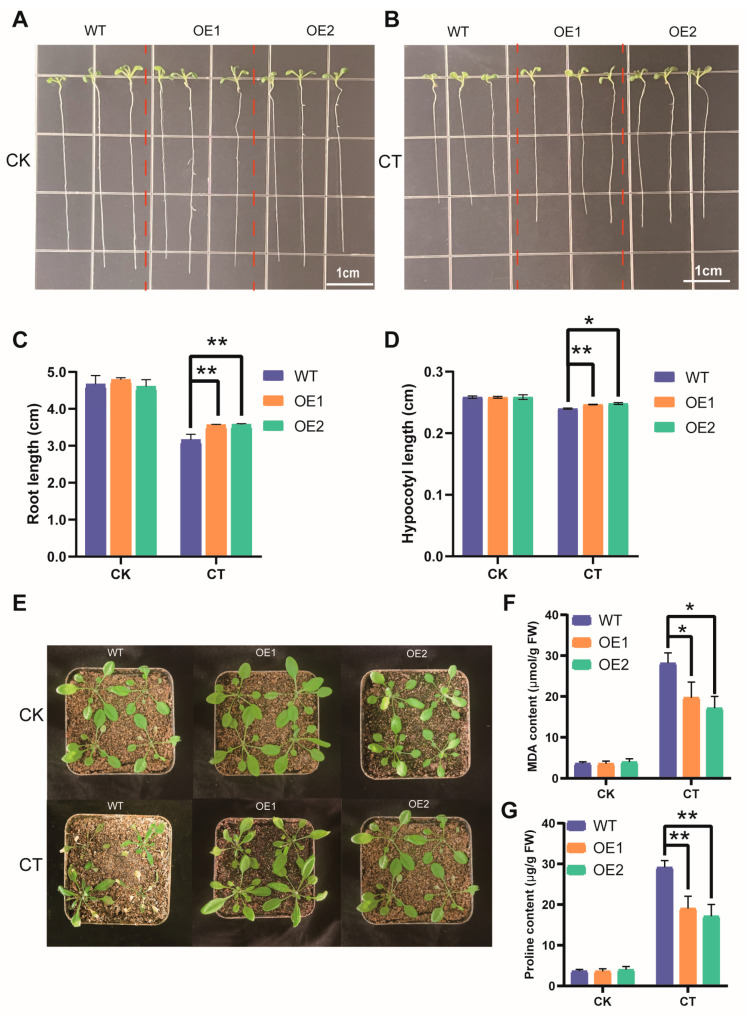
Comparison of root and hypocotyl growth of the two *AmNAC24* overexpression lines and WT plants under cold stress. (**A**) WT, *AmNAC24 OE1*, and *AmNAC24 OE2* plants cultured at normal conditions. (**B**) WT, *AmNAC24 OE1*, and *AmNAC24 OE2* plants under cold stress. (**C**) Root length of WT, *AmNAC24 OE1*, and *AmNAC24 OE2* plants in CK and CT. (**D**) Hypocotyl length of WT, *AmNAC24 OE1*, and *AmNAC24 OE2* plants in CK and CT. (**E**) Phenotypes of WT, *AmNAC24 OE1*, and *AmNAC24 OE2* plants under normal conditions and 24 h of cold stress at 4 °C. (**F**) MDA content of WT, *AmNAC24 OE1*, and *AmNAC24 OE2* plants measured under cold conditions. (**G**) Proline content of WT, *AmNAC24 OE1*, and *AmNAC24 OE2* plants measured under cold conditions. Each experiment was performed in three independent biological replicates. Values are expressed as mean ± standard deviation, and the LSD method was used to evaluate the significant difference, * *p* < 0.05, ** *p* < 0.01.

To investigate the function of *AmNAC24* in the tolerance of plants to cold stress, the phenotypic differences between the WT plants and the two transgenic lines (*AmNAC24 OE1* and *AmNAC24 OE2*) were observed at the normal culturing temperature (22 °C) and under cold stress (15 °C). At the normal culturing temperature (22 °C), plants of the WT and the two transgenic lines showed similar growth performance (Figure 9A). Under cold stress, the WT plants and transgenic lines (*OE1* and *OE2*) grew more slowly than the seedlings grown at the normal culturing temperature (Figure 9B). However, roots and hypocotyls were longer in transgenic lines (*OE1* and *OE2*) compared to WT plants under cold stress (Figure 9C,D). We also conducted low-temperature tolerance experiments using 1-month-old *Arabidopsis* plants. After cold treatment, the leaves of the WT plants displayed conspicuous wilting, whereas the frost resistance of the two transgenic lines was significantly improved, and their leaves did not exhibit noticeable wilting (Figure 9E). The MDA and proline contents increased in both overexpressed plants and WT plants. Notably, the MDA content in transgenic *Arabidopsis* leaves was significantly lower than in the WT, while the proline content in the transgenic *Arabidopsis* leaves was significantly higher (Figure 9F,G).

### 3.11. Arabidopsis Seedlings Overexpressing AmNAC24 Inhibit Excessive ROS Elevation by Activating Antioxidant Enzymes under Stress

**Figure 10:**
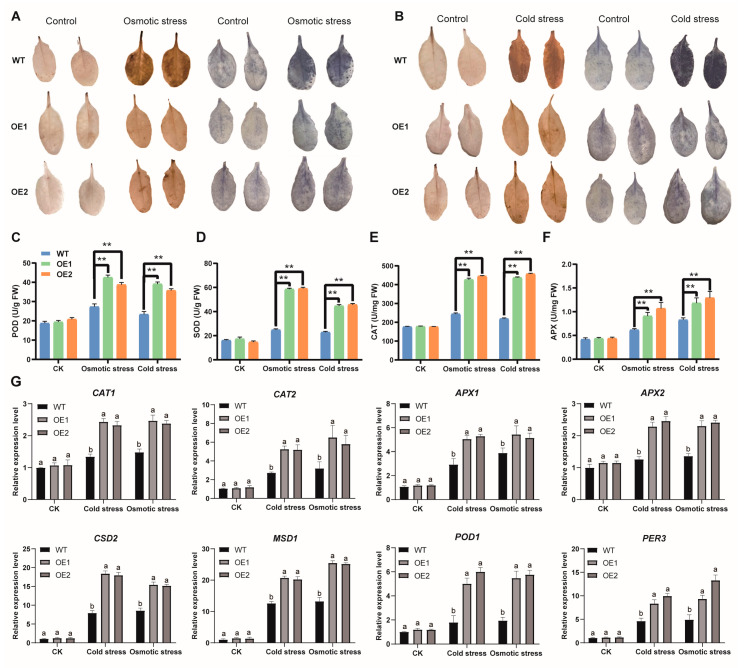
Histochemical staining with DAB and NBT and the measurement of activities of antioxidant enzymes under osmotic stress and cold stress. (**A**) H_2_O_2_ and O^2−^ accumulation was detected in the leaves of WT, *AmNAC24 OE1*, and *AmNAC24 OE2* plants under osmotic stress by using histochemical staining with DAB and NBT, respectively. The darker the orange color is, the more H_2_O_2_ accumulates. The darker the blue is, the more O^2−^ accumulates. (**B**) H_2_O_2_ and O^2−^ accumulation was detected in the leaves of WT, *AmNAC24 OE1*, and *AmNAC24 OE2* plants under cold stress by using histochemical staining with DAB and NBT, respectively. The darker the orange color is, the more H_2_O_2_ accumulates. The darker the blue is, the more O^2−^ accumulates. (**C**–**F**) Peroxidase (POD), superoxide dismutase (SOD), catalase (CAT), and ascorbate peroxidase (APX) activities of AmNAC24 overexpression lines and WT plants after the osmotic stress and cold stress treatments. Each experiment was performed in three independent biological replicates. Values are expressed as mean ± standard deviation, and the LSD method was used to evaluate the significant difference, ** *p* < 0.01. (**G**) Expression levels of eight genes related to ROS activity in WT, *AmNAC24 OE1*, and *AmNAC24 OE2* after the osmotic stress and cold stress treatments. The statistical significance is marked with lowercase letters; means with the same letters are non-significantly different for WT plants and different transgenic lines.

After the osmotic stress and cold stress treatments, the staining intensity of DAB and NBT in the leaves of the two *AmNAC24* overexpression lines and WT plants was increased, indicating that more ROS accumulated in the leaves. However, compared with the WT plants, the two *AmNAC24* overexpression lines exhibited lighter staining (Figure 10A,B).

The activities of the enzymes SOD, POD, CAT, and APX, which are involved in ROS scavenging, were analyzed. Under normal growth conditions, there was no significant difference in the activities of SOD, POD, CAT, and APX between the WT plants and the two *AmNAC24-OE* lines (Figure 10C–F). After the osmotic and cold stress treatments, the activities of SOD, POD, CAT, and APX in the *AmNAC24-OE1*, *AmNAC24-OE2*, and WT plants were significantly increased (Figure 10C–F). However, compared to the WT plants, the increase in enzyme activity of the *AmNAC24-OE1* and *AmNAC24-OE2* lines was significantly higher, and the expression of genes related to ROS activity was significantly up-regulated (Figure 10G). Taken together, these results indicate that transgenic plants overexpressing the *AmNAC24* gene may control excessive ROS elevation by increasing the activity of antioxidant enzymes.

## 4. Discussion

The uplift of the Himalayas stands as one of the most remarkable geological events in Earth’s history, exerting a profound influence on the climate and ecosystem of the Asian continent [51]. This geological transformation not only altered the climate patterns within the affected regions, but also compelled local plant populations to evolve distinct adaptation mechanisms to navigate increasingly arid and cold environmental conditions [52]. *A. mongolicus*, predominantly thriving in arid and semi-arid regions across Asia, exemplifies a species tailored to this climatic evolution. The harsh environment has propelled the evolutionary trajectory of *A. mongolicus*, leading to the acquisition of significant traits such as drought and cold resistance [53]. Studies have demonstrated the remarkable cold and drought tolerance of *A. mongolicus*, showcasing its adaptability to desert environments [54,55]. NAC proteins, a class of plant-specific TFs, are crucial for plant adaptation to a variety of biotic and abiotic stresses [56]. Despite extensive investigations on the NAC gene family in model species like rice [26], *Arabidopsis* [25], and tobacco [57] owing to advancements in genome sequencing technology, research on this gene family in non-model species, particularly desert plants, remains inadequate. This study systematically examines the structure and evolutionary characteristics of the NAC gene family in *A. mongolicus*, shedding light on *AmNAC24* as a potential candidate gene involved in osmotic and cold stress responses through functional analysis.

The quantity of NAC family members within a specific plant species not only correlates with the size of the genome of the species, but also reflects the enduring impact of long-term plant evolution. Here, 86 NAC proteins were identified in *A. mongolicus*. Compared with the genomes of other species, the count of NAC genes in other species such as *M. sativa* [58], *Glycine max* [59], *Dendrobium officinale* [60], and *Zanthoxylum bungeanum* [29] stands at 421, 269, 111, and 109, respectively. *A. mongolicus* exhibits a relatively modest count of NAC family members compared to these plants, potentially indicative of evolutionary adaptations in distinct species amid varying habitat conditions. Whole-genome duplication stands as the principal driving force in plant genome evolution, prominently manifesting through segmental and tandem duplications within gene families [61]. The identification of 34 pairs of segmental duplications and 4 pairs of tandem duplications in *A. mongolicus* emphasizes that segmental duplication is the principal evolutionary force shaping the AmNAC family. This phenomenon has been documented in studies exploring NAC families of *Isatis indigotica* [62], *Panicum miliaceum* [63], and *Capsicum annuum* [28]. Analysis based on KaKs calculations reveals that all duplicate gene pairs evolved under purifying selection, indicating that *A. mongolicus* has become well adapted to extreme environments of cold and drought through long-term evolution.

Phylogenetic analysis showed that AmNACs are divided into 15 groups, which is like the NAC family in *Isatis indigotica* [62], but different from *Kandelia bovate* (16 groups) [64] and *Dimocarpus longan* (14 groups) [65]. The NAC family is categorized into three subfamilies: NAM, ATAF, and CUC. The functional annotation of the *AmNAC* gene in *A. mongolicus* was established based on its homologous relationship with the NAC family gene in *A. thaliana*. Among the 15 groups constituting the *A. mongolicus* NAC family, the NAM subfamily boasts the highest membership. Prior studies underscored the multifaceted roles of the NAM subfamily, encompassing plant development, stress response, immunity, and metabolism, signifying its pivotal role in plant environmental stress adaptation [8]. The presence and count of introns offer insights into genome complexity and evolutionary processes [66]. In the evolution of NAC family genes, intron addition, loss, or recombination may engender novel functions or alterations in existing functions. The introns within the NAC family members of *A. mongolicus* range between 1–8, akin to the NAC gene family (1–6) in *Cicer arietinum* [67], also a legume plant. The integration of conserved domain motifs revealed closely related AmNAC members sharing analogous introns and conserved motifs, hinting at potentially similar biological functions.

The promoter regions of *NAC* genes contain stress-responsive elements that can bind to specific stress-responsive TFs, thereby initiating or inhibiting the transcription of *NAC* genes [8]. These stress-responsive elements include ABREs (ABA-responsive elements), DREs (drought-responsive elements), and LTREs (low-temperature-responsive elements). Analyzing the expression patterns of *AmNAC* genes from summer to winter, it was observed that the *AmNAC* genes exhibiting either a consistent increase or decrease in expression levels all contained at least one of these cis-acting elements: ABREs, DREs, or LTREs. This suggests that cis-regulatory elements play crucial roles in modulating the expression of genes responsive to specific environmental stresses, aiding the plant’s adaptation to challenging conditions such as low temperatures and drought stresses.

Research indicates the involvement of multiple members within the ATAF subfamily in plant abiotic stress responses [68]. The overexpression of *OsNAC5* in rice has demonstrated an augmentation in plant stress resistance, contributing to enhanced rice yield [20]. Within *A. mongolicus*, there are eight AmNAC members belonging to the ATAF subfamily, wherein AmNAC24 and AmNAC81 cluster alongside ANAC019, ANAC055, and ANAC072 within a distinct branch in the phylogenetic tree. Leveraging RNA-seq data analysis and qRT-PCR, this study discerned the key *AmNAC* genes pivotal in two stress responses. Expression analysis underscores the involvement of a substantial number of NAC members in responding to osmotic stress and cold stress. Notably, under osmotic stress conditions, the *AmNAC24*, *AmNAC37*, and *AmNAC57* genes from the ATTF subfamily exhibit heightened expression patterns, which persist similarly under cold stress. These findings strongly implicate the crucial roles these genes might play in the response of *A. mongolicus* to various abiotic stresses.

To investigate the role of the ATAF subfamily members in *A. mongolicus*, the *AmNAC24* gene, whose expression significantly increased under cold and osmotic stress, was isolated and functionally characterized. Arabidopsis plants transformed with *AmNAC24* exhibited enhanced growth under osmotic and cold stress compared to the wild type. The *AmNAC24* gene may be a key gene that plays a regulatory role in adaptation to osmotic and cold stress for *A. mongolicus*. Homologs of *AmNAC24* in *Arabidopsis*, namely *ANAC019*, *ANAC055*, and *ANAC072*, have been reported to play significant roles in responding to abiotic stresses. The overexpression of these genes has been observed in increased drought tolerance in transgenic plants [17]. Additionally, in tobacco, the overexpression of the *NtNAC053* gene, the homolog of *AmNAC24*, enhanced the tolerance of tobacco to salt and drought stress [69].

ROS homeostasis is crucial for plant growth, development, and adaptation to stress. Drought and cold stress can lead to the excessive accumulation of ROS in plants, thereby damaging their normal growth and development [70]. In the present study, under osmotic and cold stress, the DAB and NBT staining of *AmNAC24*-overexpressing transgenic *Arabidopsis* was lighter than that of wild-type plants, indicating that the transgenic lines had a stronger ability to scavenge reactive oxygen species than wild-type plants. The activity detection of the antioxidant enzymes SOD, POD, CAT, and APX revealed that, compared with the wild type, the transgenic plants showed a significant increase in SOD, POD, CAT, and APX activity, indicating that the overexpression of *AmNAC24* improved the ability of transgenic *Arabidopsis* to scavenge reactive oxygen species. Similar results have also been reported in other plants; for example, under drought stress, the DAB and NAT staining of leaves in *Camellia sinensis NAC28* [71]-overexpressing *Arabidopsis* plants is lighter than those in the wild type, indicating that *Arabidopsis* plants overexpressing the *CsNAC28* gene can reduce the accumulation of ROS under stress conditions. Under salt and drought stress, the CAT, POD, and SOD activities of tobacco seedlings overexpressing the tobacco *NAC053* gene were significantly higher than those of wild-type plants [69]. We speculate that the *AmNAC24* gene may enhance the activity of the antioxidant enzymes SOD, POD, CAT, and APX by affecting their expression, thereby better inhibiting the excessive increase in ROS levels under osmotic and cold stress in transgenic *Arabidopsis*.

## 5. Conclusions

In the present study, a comprehensive analysis was conducted on the AmNAC members of *A. mongolicus*, covering their distribution on chromosomes, basic physicochemical characteristics, phylogenetic classification, gene structure, and evolutionary history. Gene expression analysis shows that the expression levels of many members of the AmNAC family changed under osmotic stress and cold stress, suggesting that these genes may be involved in the environmental response of *A. mongolicus*. The overexpression of a cold and osmotic-stress-induced NAC family member gene, *AmNAC24*, improved the tolerance of transgenic *Arabidopsis* plants under cold stress and osmotic stress. This study advanced our comprehension of the biological function of the NAC gene in *A. mongolicus* and offered crucial data for elucidating the mechanism associated with the stress resistance of *A. mongolicus*.

The response of plants to abiotic stress is a complex regulatory network, and the study of the NAC genes involved in the response of non-model species such as *A. mongolicus* to biotic stress can provide important data for a comprehensive analysis of plant response mechanisms to environmental stress. Further research is needed to identify the specific upstream and downstream genes and to determine the action mechanism of the *AmNAC24* genes that significantly regulate osmotic and cold stress; this is to provide more evidence for understanding the *NAC* gene response of *A. mongolicus* to abiotic stress.

## Data Availability

All data supporting this study are available within the paper and within the Supplementary Materials published online.

## Supplementary Materials

The following supporting information can be downloaded at: https://www.mdpi.com/article/10.3390/biom14020182/s1, Figure S1: Multiple sequence alignment of AmNAC proteins. Red, blue, and green represent sequences with a conservative proportion of 90%, 80%, and 60%, respectively, and the black wireframes represent the five motifs of AmNAC proteins (Motif A–E); Figure S2: Conserved motif and gene structures of the *A. mongolicus* NAC family genes. (A) The sequence logos of the detected motifs. The height of each letter indicates the degree of conservation. Numbers on the *x*-axis represent residue positions, and the *y*-axis represents the content measured in bits. (B) Conserved motifs in the 86 AmNAC proteins, different motifs are presented in different colored boxes. (C) Gene structures of *AmNAC* genes. Yellow boxes represent untranslated regions (UTR), and green boxes represent coding sequence (CDS); Figure S3: Multicollinearity analysis of *NAC* genes between *A. mongolicus* and *V. vinifera*, *A. thaliana*, *M. truncatula*, and *L. albus*, the orange line represents the collinearity of the *NAC* gene of *A. mongolicus* and other genomes. Table S1: The sequences of primers for qRT-PCR analysis; Table S2: Physicochemical properties and predicted subcellular localization of 86 AmNAC members; Table S3: Ka/Ks of *AmNAC* gene paralogous pairs in *A. mongolicus*.

## References

[1] Lesk C., Rowhani P., Ramankutty N. (2016). Influence of extreme weather disasters on global crop production. Nature.

[2] Lobell D.B., Schlenker W., Costa-Roberts J. (2011). Climate trends and global crop production since 1980. Science.

[3] Hossain M.A., Bhattacharjee S., Armin S.M., Qian P., Xin W., Li H.Y., Burritt D.J., Fujita M., Tran L.S. (2015). Hydrogen peroxide priming modulates abiotic oxidative stress tolerance: Insights from ROS detoxification and scavenging. Front. Plant Sci..

[4] Jogaiah S., Abdelrahman M., Tran L.P., Ito S.I. (2018). Different mechanisms of Trichoderma virens-mediated resistance in tomato against Fusarium wilt involve the jasmonic and salicylic acid pathways. Mol. Plant Pathol..

[5] Javed T., Shabbir R., Ali A., Afzal I., Zaheer U., Gao S.J. (2020). Transcription Factors in Plant Stress Responses: Challenges and Potential for Sugarcane Improvement. Plants.

[6] Manna M., Thakur T., Chirom O., Mandlik R., Deshmukh R., Salvi P. (2021). Transcription factors as key molecular target to strengthen the drought stress tolerance in plants. Physiol. Plant.

[7] Nakashima K., Takasaki H., Mizoi J., Shinozaki K., Yamaguchi-Shinozaki K. (2012). NAC transcription factors in plant abiotic stress responses. Biochim. Biophys. Acta.

[8] Han K., Zhao Y., Sun Y., Li Y. (2023). NACs, generalist in plant life. Plant Biotechnol. J..

[9] Li W., Pang S., Lu Z., Jin B. (2020). Function and Mechanism of WRKY Transcription Factors in Abiotic Stress Responses of Plants. Plants.

[10] Li C., Ng C.K.Y., Fan L.M. (2015). MYB transcription factors, active players in abiotic stress signaling. Environ. Exp. Bot..

[11] Souer E., van Houwelingen A., Kloos D., Mol J., Koes R. (1996). The no apical meristem gene of Petunia is required for pattern formation in embryos and flowers and is expressed at meristem and primordia boundaries. Cell.

[12] Aida M., Ishida T., Fukaki H., Fujisawa H., Tasaka M. (1997). Genes involved in organ separation in Arabidopsis: An analysis of the cup-shaped cotyledon mutant. Plant Cell.

[13] Olsen A.N., Ernst H.A., Leggio L.L., Skriver K. (2005). DNA-binding specificity and molecular functions of NAC transcription factors. Plant Sci..

[14] Diao P., Chen C., Zhang Y., Meng Q., Lv W., Ma N. (2020). The role of NAC transcription factor in plant cold response. Plant Signal Behav..

[15] Srivastava R. (2021). Understanding NAC Transcription Factor Mediated Signaling in the Regulation of Growth and Abiotic Stress Tolerance in Cowpea.

[16] Wang J., Wang Y., Zhang J., Ren Y., Li M., Tian S., Yu Y., Zuo Y., Gong G., Zhang H. (2021). The NAC transcription factor ClNAC68 positively regulates sugar content and seed development in watermelon by repressing ClINV and ClGH3.6. Hortic. Res..

[17] Bu Q., Jiang H., Li C.B., Zhai Q., Zhang J., Wu X., Sun J., Xie Q., Li C. (2008). Role of the *Arabidopsis thaliana* NAC transcription factors ANAC019 and ANAC055 in regulating jasmonic acid-signaled defense responses. Cell Res..

[18] Jeong J.S., Kim Y.S., Baek K.H., Jung H., Ha S.H., Do Choi Y., Kim M., Reuzeau C., Kim J.K. (2010). Root-specific expression of OsNAC10 improves drought tolerance and grain yield in rice under field drought conditions. Plant Physiol..

[19] Redillas M.C., Jeong J.S., Kim Y.S., Jung H., Bang S.W., Choi Y.D., Ha S.H., Reuzeau C., Kim J.K. (2012). The overexpression of OsNAC9 alters the root architecture of rice plants enhancing drought resistance and grain yield under field conditions. Plant Biotechnol. J..

[20] Jeong J.S., Kim Y.S., Redillas M.C., Jang G., Jung H., Bang S.W., Choi Y.D., Ha S.H., Reuzeau C., Kim J.K. (2013). OsNAC5 overexpression enlarges root diameter in rice plants leading to enhanced drought tolerance and increased grain yield in the field. Plant Biotechnol. J..

[21] Rachmat A., Nugroho S., Sukma D., Aswidinnoor H. (2014). Overexpression of OsNAC6 transcription factor from Indonesia rice cultivar enhances drought and salt tolerance. EMIR J. Food. AGR.

[22] Xu X., Yao X., Lu L., Zhao D. (2018). Overexpression of the transcription factor NtNAC2 confers drought tolerance in tobacco. Plant Mol. Biol. Newsl..

[23] Shah S.T., Pang C., Hussain A., Fan S., Song M., Zamir R., Yu S. (2014). Molecular cloning and functional analysis of NAC family genes associated with leaf senescence and stresses in *Gossypium hirsutum* L.. Plant Cell.

[24] Qu Y., Duan M., Zhang Z., Dong J., Wang T. (2016). Overexpression of the Medicago falcata NAC transcription factor MfNAC3 enhances cold tolerance in *Medicago truncatula*. Environ. Exp. Bot..

[25] Ooka H., Satoh K., Doim K., Nagata T., Otomo Y., Murakami K., Matsubara K., Osato N., Kawai J., Carninci P. (2003). Comprehensive analysis of NAC family genes in *Oryza sativa* and *Arabidopsis thaliana*. DNA Res..

[26] Wang G., Yuan Z., Zhang P., Liu Z., Wang T., Wei L. (2020). Genome-wide analysis of NAC transcription factor family in maize under drought stress and rewatering. Physiol. Mol. Biol. Plants.

[27] Tariq R., Hussain A., Tariq A., Khalid M.H.B., Khan I., Basim H., Ingvarsson P.K. (2022). Genome-wide analyses of the mung bean NAC gene family reveals orthologs, co-expression networking and expression profiling under abiotic and biotic stresses. BMC Plant Biol..

[28] Diao W., Snyder J.C., Wang S., Liu J., Pan B., Guo G., Ge W., Dawood M.H.S.A. (2018). Genome-wide analyses of the NAC transcription factor gene family in pepper (*Capsicum annuum* L.): Chromosome location, phylogeny, structure, expression patterns, cis-elements in the promoter, and interaction network. Int. J. Mol. Sci..

[29] Hu H., Ma L., Chen X., Fei X., He B., Luo Y., Liu Y., Wei A. (2022). Genome-wide identification of the NAC gene family in *Zanthoxylum bungeanum* and their transcriptional responses to drought stress. Int. J. Mol. Sci..

[30] Li X., He X., Hou L., Ren Y., Wang S., Su F. (2018). Dark septate endophytes isolated from a xerophyte plant promote the growth of *Ammopiptanthus mongolicus* under drought condition. Sci. Rep..

[31] Zhou Y., Gao F., Liu R., Feng J., Li H. (2012). De novo sequencing and analysis of root transcriptome using 454 pyrosequencing to discover putative genes associated with drought tolerance in *Ammopiptanthus mongolicus*. BMC Genom..

[32] Wu Y., Wei W., Pang X., Wang X., Zhang H., Dong B., Xing Y., Li X., Wang M. (2014). Comparative transcriptome profiling of a desert evergreen shrub, *Ammopiptanthus mongolicus*, in response to drought and cold stresses. BMC Genom..

[33] Gao F., Wang J., Wei S., Li Z., Wang N., Li H., Feng J., Li H., Zhou Y., Zhang F. (2015). Transcriptomic Analysis of Drought Stress Responses in *Ammopiptanthus mongolicus* Leaves Using the RNA-Seq Technique. PLoS ONE.

[34] Gao F., Wang N., Li H., Liu J., Fu C., Xiao Z., Wei C., Lu X., Feng J., Zhou Y. (2016). Identification of drought-responsive microRNAs and their targets in *Ammopiptanthus mongolicus* by using high-throughput sequencing. Sci. Rep..

[35] Shi J., Liu M., Chen Y., Wang J., Lu C. (2016). Heterologous expression of the dehydrin-like protein gene *AmCIP* from *Ammopiptanthus mongolicus* enhances viability of Escherichia coli and tobacco under cold stress. Plant Growth Regul..

[36] Ren M., Wang Z., Xue M., Wang X., Zhang F., Zhang Y., Zhang W., Wang M. (2019). Constitutive expression of an A-5 subgroup member in the DREB transcription factor subfamily from *Ammopiptanthus mongolicus* enhanced abiotic stress tolerance and anthocyanin accumulation in transgenic *Arabidopsis*. PLoS ONE.

[37] Pang X., Xue M., Ren M., Nan D., Wu Y., Guo H. (2019). *Ammopiptanthus mongolicus* stress-responsive NAC gene enhances the tolerance of transgenic *Arabidopsis thaliana* to drought and cold stresses. Genet. Mol. Biol..

[38] Tang K., Zhang Y., Ren M., Xue M., Zhang M., Pang X., Wang M. (2023). Constitutive expression of a membrane-bound NAC transcription factor AmNTL1 from desert shrub *Ammopiptanthus mongolicus* enhances abiotic stress tolerance of transgenic *Arabidopsis*. J. S. Afr. Bot..

[39] Mistry J., Finn R.D., Eddy S.R., Bateman A., Punta M. (2013). Challenges in homology search: HMMER3 and convergent evolution of coiled-coil regions. Nucleic Acids Res..

[40] Chen C., Wu Y., Li J., Wang X., Zeng Z., Xu J., Liu Y., Feng J., Chen H., He Y. (2023). TBtools-II: A “one for all, all for one” bioinformatics platform for biological big-data mining. Mol. Plant.

[41] Edgar R.C. (2004). MUSCLE: Multiple sequence alignment with high accuracy and high throughput. Nucleic Acids Res..

[42] Bailey T.L., Johnson J., Grant C.E., Noble W.S. (2015). The MEME suite. Nucleic Acids Res..

[43] Lescot M., Déhais P., Thijs G., Marchal K., Moreau Y., Van de Peer Y., Rouzé P., Rombauts S. (2002). PlantCARE, a database of plant cis-acting regulatory elements and a portal to tools for in silico analysis of promoter sequences. Nucleic Acids Res..

[44] Wang Y., Tang H., DeBarry J.D., Tan X., Li J., Wang X., Lee T.H., Jin H., Marler B., Guo H. (2012). MCScanX: A toolkit for detection and evolutionary analysis of gene synteny and collinearity. Nucleic Acids Res..

[45] Wang D., Zhang Y., Zhang Z., Zhu J., Yu J. (2010). KaKs_Calculator 2.0: A toolkit incorporating gamma-series methods and sliding window strategies. Genom. Proteom. Bioinf..

[46] Trapnell C., Pachter L., Salzberg S.L. (2009). TopHat: Discovering splice junctions with RNA-Seq. Bioinformatics.

[47] Wang Y., Cao S., Sui X., Wang J., Geng Y., Gao F., Zhou Y. (2022). Genome-wide characterization, evolution, and expression analysis of the ascorbate peroxidase and glutathione peroxidase gene families in response to cold and osmotic stress in *Ammopiptanthus nanus*. J. Plant Growth Regul..

[48] Abla M., Sun H., Li Z., Wei C., Gao F., Zhou Y., Feng J. (2019). Identification of miRNAs and their response to cold stress in *Astragalus membranaceus*. Biomolecules.

[49] Kumar P., Tewari R.K., Sharma P.N. (2008). Cadmium enhances generation of hydrogen peroxide and amplifies activities of catalase, peroxidases and superoxide dismutase in maize. J. Agron. Crop Sci..

[50] Kumar D., Yusuf M.A., Singh P., Sardar M., Sarin N.B. (2014). Histochemical detection of superoxide and H_2_O_2_ accumulation in *Brassica juncea* seedlings. Bio-Protocol.

[51] Tada R., Zheng H., Clift P.D. (2016). Evolution and variability of the Asian monsoon and its potential linkage with uplift of the Himalaya and Tibetan Plateau. Prog. Earth Planet. Sci..

[52] Burbank D.W. (1992). Causes of recent Himalayan uplift deduced from deposited patterns in the Ganges basin. Nature.

[53] Jiang S., Luo M.X., Gao R.H., Zhang W., Yang Y.Z., Li Y.J., Liao P.C. (2019). Isolation-by-environment as a driver of genetic differentiation among populations of the only broad-leaved evergreen shrub *Ammopiptanthus mongolicus* in Asian temperate deserts. Sci. Rep..

[54] Cui H., Wang Y., Yu T., Chen S., Chen Y., Lu C. (2020). Heterologous expression of three *Ammopiptanthus mongolicus* dehydrin genes confers abiotic stress tolerance in *Arabidopsis thaliana*. Plants.

[55] Li X., Liu Q., Wu R., Bing J., Zheng L., Sumbur B., Zhou Y., Gao F. (2023). Proteomic analysis of the cold stress response of *Ammopiptanthus mongolicus* reveals the role of AmCHIA in its cold tolerance. Horticulturae.

[56] Shao H., Wang H., Tang X. (2015). NAC transcription factors in plant multiple abiotic stress responses: Progress and prospects. Front. Plant Sci..

[57] Li W., Li X., Chao J., Zhang Z., Wang W., Guo Y. (2018). NAC family transcription factors in tobacco and their potential role in regulating leaf senescence. Front. Plant Sci..

[58] He F., Zhang L., Zhao G., Kang J., Long R., Li M., Yang Q., Chen L. (2022). Genome-wide identification and expression analysis of the NAC gene family in Alfalfa revealed its potential roles in response to multiple abiotic stresses. Int. J. Mol. Sci..

[59] Pinheiro G.L., Marques C.S., Costa M.D., Reis P.A., Alves M.S., Carvalho C.M., Fietto L.G., Fontes E.P. (2009). Complete inventory of soybean NAC transcription factors: Sequence conservation and expression analysis uncover their distinct roles in stress response. Gene.

[60] Yang Q., Li Z., Wang X., Jiang C., Liu F., Nian Y., Fu X., Zhou G., Liu L., Wang H. (2023). Genome-wide identification and characterization of the NAC gene family and its involvement in cold response in dendrobium officinale. Plants.

[61] Chauve C., Doyon J.P., El-Mabrouk N. (2008). Gene family evolution by duplication, speciation, and loss. J. Comput. Biol..

[62] Wang Z., Zhang Z., Wang P., Qin C., He L., Kong L., Ren W., Liu X., Ma W. (2023). Genome-wide identification of the NAC transcription factors family and regulation of metabolites under salt stress in *Isatis indigotica*. Int. J. Biol. Macromol..

[63] Shan Z., Jiang Y., Li H., Guo J., Dong M., Zhang J., Liu G. (2020). Genome-wide analysis of the NAC transcription factor family in broomcorn millet (*Panicum miliaceum* L.) and expression analysis under drought stress. BMC Genom..

[64] Sun M.M., Liu X., Huang X.J., Yang J.J., Qin P.T., Zhou H., Jiang M.G., Liao H.Z. (2022). Genome-wide identification and expression analysis of the NAC gene family in *Kandelia obovata*, a typical mangrove plant. Curr. Issues Mol. Biol..

[65] Munir N., Yukun C., Xiaohui C., Nawaz M.A., Iftikhar J., Rizwan H.M., Xu S., Yuling L., Xuhan X., Zhongxiong L. (2020). Genome-wide identification and comprehensive analyses of NAC transcription factor gene family and expression patterns during somatic embryogenesis in *Dimocarpus longan* Lour. Plant Physiol. Biochem..

[66] Poverennaya I.V., Roytberg M.A. (2020). Spliceosomal Introns: Features, Functions, and Evolution. Biochemistry.

[67] Ha C.V., Esfahani M.N., Watanabe Y., Tran U.T., Sulieman S., Mochida K., Nguyen D.V., Tran L.S. (2014). Genome-wide identification and expression analysis of the CaNAC family members in chickpea during development, dehydration and ABA treatments. PLoS ONE.

[68] Christianson J.A., Dennis E.S., Llewellyn D.J., Wilson I.W. (2010). ATAF NAC transcription factors: Regulators of plant stress signaling. Plant Signal Behav..

[69] Li X., Wang Q., Guo C., Sun J., Li Z., Wang Y., Yang A., Pu W., Guo Y., Gao J. (2022). NtNAC053, a novel NAC transcription factor, confers drought and salt tolerances in tobacco. Front. Plant Sci..

[70] Ray P.D., Huang B.W., Tsuji Y. (2012). Reactive oxygen species (ROS) homeostasis and redox regulation in cellular signaling. Cell Signal.

[71] Zhang X., Li L., Lang Z., Li D., He Y., Zhao Y., Tao H., Wei J., Li Q., Hong G. (2022). Genome-wide characterization of NAC transcription factors in *Camellia sinensis* and the involvement of *CsNAC28* in drought tolerance. Front. Plant Sci..

