# Supplementary figures and images for: Characterization of NAC Gene Family in Ammopiptanthus mongolicus and Functional Analysis of AmNAC24, an Osmotic and Cold-Stress-Induced NAC Gene
Source: Biomolecules. 2024 Feb 2;14(2):182. doi: 10.3390/biom14020182 (PMC10886826; doi:10.3390/biom14020182)

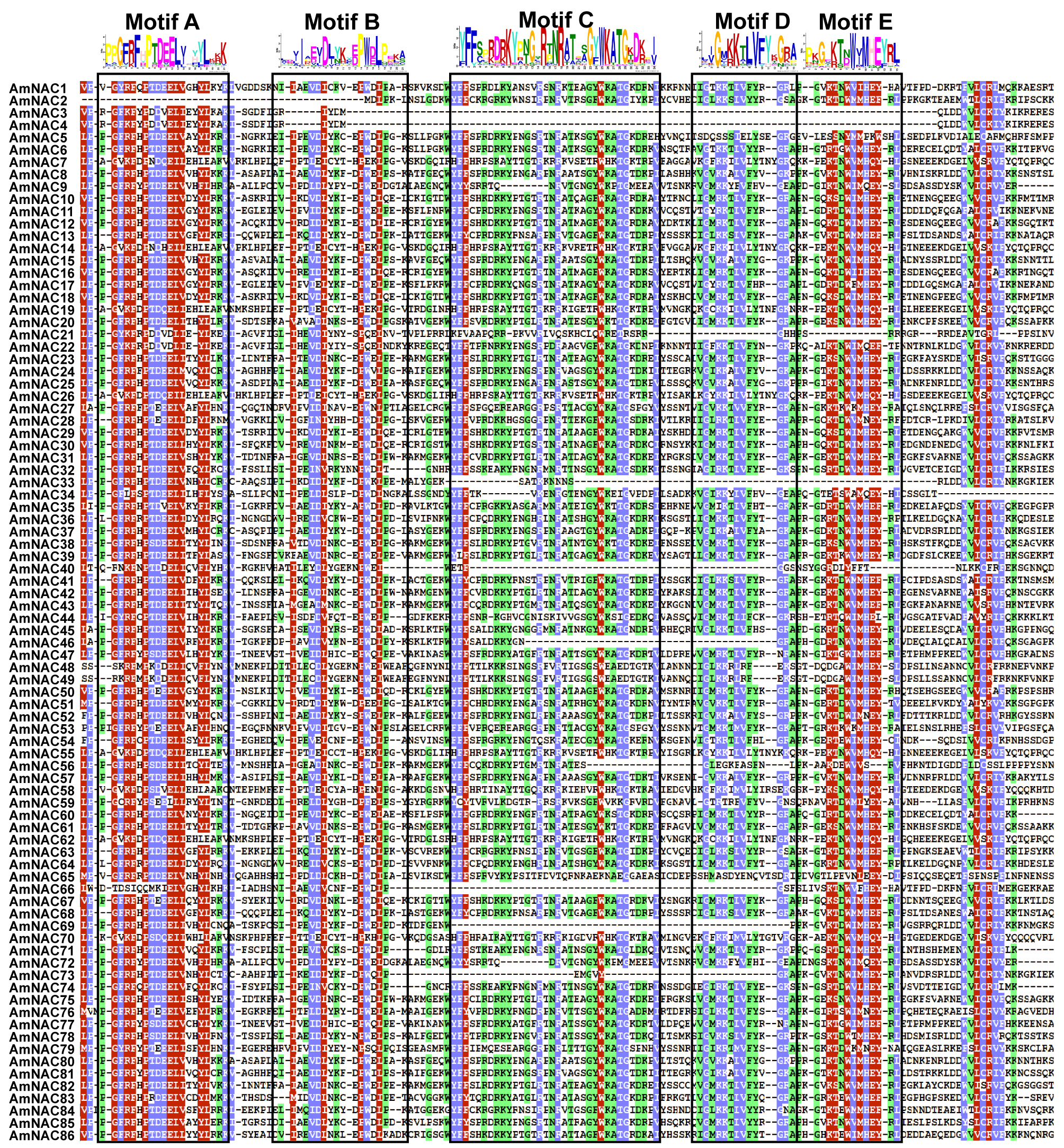

Supplement: Supplementary file 1 [file biomolecules-14-00182-s001.zip › FigureS1.tif]

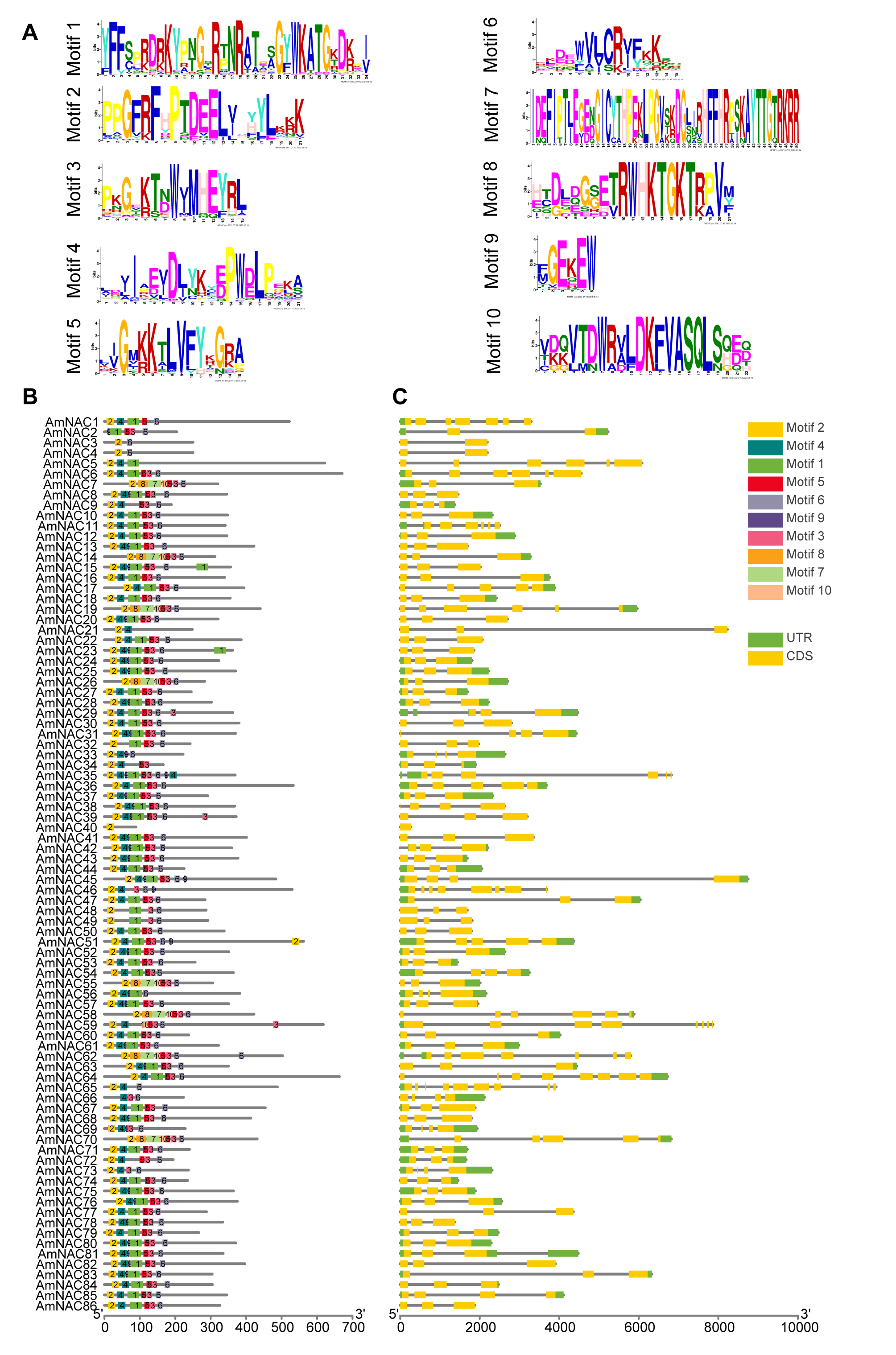

Supplement: Supplementary file 1 [file biomolecules-14-00182-s001.zip › FigureS2.tif]

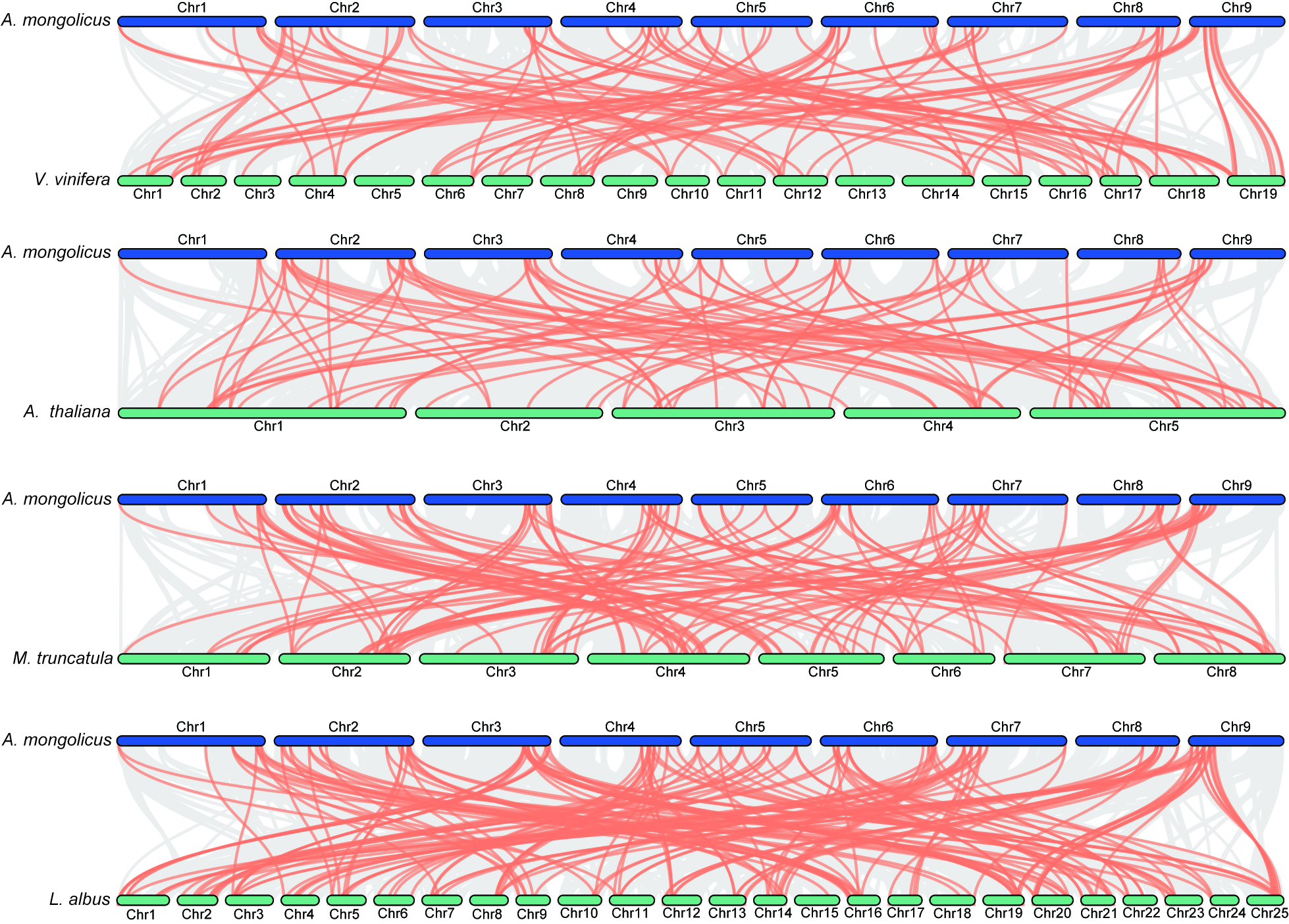

Supplement: Supplementary file 1 [file biomolecules-14-00182-s001.zip › FigureS3.tif]
